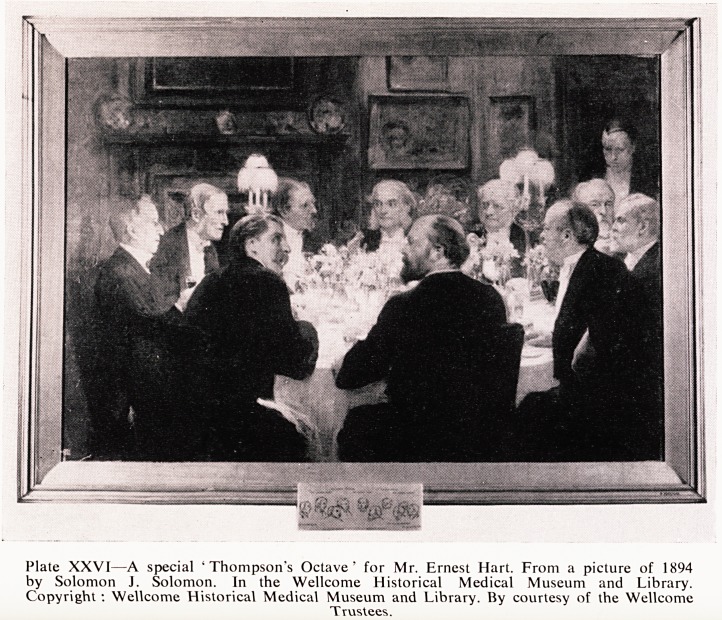# Louis Napoleon and His Doctors

**Published:** 1967-07

**Authors:** S. F. Marwood


### LOUIS NAPOLEON AND HIS DOCTORS

BY

S. F. MARWOOD, M.D., M.R.C.P.

*Presidential address to the Bristol Medico-Chirurgical Society*
*October 12th, 1966*

Royalty has always surrounded itself with doctors for reasons which are obvious and have usually, though not invariably, proved to be justifiable; and doctors for the most part have served their royal patients faithfully and well, and often indeed with great devotion. To-night I propose, necessarily briefly, to tell the story of an illustrious patient and of some of the doctors who attended him. The patient was Charles Louis Bonaparte, later and better known as Louis Napoleon, who may not unfairly be described as both founder and destroyer of the French Second Empire over which he reigned for some two decades as the Emperor Napoleon the Third with dictatorial powers.

To most of us here in England, this emperor is a somewhat shadowy figure who is remembered chiefly for the disasters of the Franco-German war and for his capture by the Germans at Sedan. Inevitably he has suffered by comparison with his famous uncle, the great Napoleon Bonaparte, and he has been dismissed as a weak and irresolute man of small capacity, and even been dubbed Napoleon the Little. To live in the shadow of genius is no easy situation for any man, and Louis Napoleon was certainly no genius, but that is not to say that he was the incompetent weakling he is so often represented to be.

We are not concerned here with his earlier years, and it will suffice for me to say that he was born in 1808 the son of Joseph, King of Holland and a brother of the great Emperor, and that he was eventually brought up by his mother in exile in Switzerland. On his reaching manhood, contemporary accounts are of an attractive, intelligent, well-informed and athletic young man. Thenceforward he led an exciting and adventurous life during which, following an abortive uprising, he was imprisoned for life in a forbidding French fortress from which he escaped only after six years' incarceration. He wrote widely and well on a variety of subjects and by various means successfully kept alive and fostered the Napoleonic legend in France. His constant aim and objective were the restoration of the Empire, and against great odds he succeeded. It was an achievement which may not have required genius, but which certainly called for qualities of intellect, courage, and resolution, as well as fortitude in the face of many disappointments. To all these attributes many reputable observers have testified. Simpson, the Cambridge historian of the earlier years of this century, says " Louis Napoleon's career provided one of the most striking examples which history can afford of the mountain-moving force of faith, and the skill, without which faith alone is useless, was his also ".

That in later life he did display weakness and irresolution there is no doubt, and I believe that these defects, which he masked behind an inscrutable demeanour, were due in no small part to the chronic debilitating and often incapacitating illness which dogged him through the greater part of his reign, and which indeed became almost unbearable in his closing years. Louis Napoleon has been described as the most famous patient in the history of urology, and he was attended by many doctors some of whom bore names which were to become famous in the annals of our profession.

The year 1848 was a fateful one for Europe. Revolution was rife and thrones were toppling. France was no exception. Louis Philippe, the last of the ancient line of French kings, was deposed, and the Second Republic was established. Louis Napoleon hastily returned to France and secured election to the Assembly, and in December of the same year he was, to the astonishment of all republicans, elected President by the overwhelming majority of the French people. He adopted the title of Prince President, and three years later he seized absolute power by a coup d'état. Thence to the imperial crown was but a step, and this he took in 1852. The following year, at the age of forty-five, he married the Spanish Countess Eugenie di Montijo, a sister of the Duchess of Alba. She was twenty-five and a reigning beauty, and she made him a loyal and dignified consort if a somewhat impetuous one, while their court became for a time the most glittering in Europe. She was to outlive him by nearly half a century.

The period of his sway may be divided approximately into two decades, the 1850's and the 1860's. The first of these has been described by Mowatt, formerly professor of history in this university of Bristol, as the happy years of the Empire. France was prosperous, and the bulk of her population was contented. She ranked high in the councils of Europe and was recognised as the world's foremost military power. Nevertheless, she was under a dictatorship. There was a ban on free speech, and of course a press censorship. And although orators and journalists with characteristic Gallic wit and ingenuity contrived partially to evade restrictions, there were limits beyond which they dared not go, and high on the list of taboos was any unauthorised reference to the Emperor's health. Throughout the reign there was at all times a conspiracy of silence in Palace circles concerning this subject, for it was believed to be harmful to the interests of France and the dynasty that the Emperor should ever be thought to be in failing health and unable to perform his duties as Head of State. The dictum of Machiavelli that " The Prince may never be ill " was taken very seriously indeed, and it will thus be understood why the various events connected with the Emperor's developing malady became generally known only towards the end of his reign, or indeed after his death, while some of the circumstances are still a matter of conjecture. It would seem however that, during this first decade, the Emperor's health though undoubtedly poor at times gave no cause for serious anxiety, and rarely interfered with his conduct of affairs. But there was one incident which, to those in the know, must have seemed ominous. In 1856 the famous London surgeon, Sir William Fergusson (Plate XXI), was sent for. Why, no one really knows, but a brief look at Fergusson's career provides a good clue.

Born in 1808, William Fergusson left a lawyer's office at the age of seventeen to become a medical student at Edinburgh, and he was still only twenty-one when elected to the Fellowship of the senior surgical college, the Royal College of Surgeons of Edinburgh. At the age of twenty-nine, he was elected surgeon to the Edinburgh Royal Infirmary and when, three years later, he was appointed to the chair of surgery at Kings College, London, he arrived with an established Scottish reputation as surgeon and

Plate IXX—Napoléon III (1808-1873) at the time of his accession. From a lithograph in the British Museum, after the painting by Alfred de Dreux.

Copyright : British Museum

Plate XX—Eugénie, Empress of the French (1826-1920). Drawn and engraved by D. J. Pound. In the Wellcome Historical Medical Museum and Library. By courtesy of the Wellcome Trustees.

anatomist. In London, he quickly became known as a brilliant operating surgeon. A friendly and generous man, he was loved by his students many of whom he helped financially and in other ways, and he was fond of entertaining them and his various friends at the old Albion Tavern in the City of London. A guest on one of these occasions was Charles Lemon, then Editor of Punch, indeed its first editor and editor for thirty years, and Lemon concluded his acceptance of the invitation characteristically in these terms, "Look out for me at seven, but look after me at eleven". To mention only a few of Fergusson's distinctions, Edinburgh University made him an honorary Ll.D., and he was elected to the Fellowship of the English Royal College of Surgeons where he delivered many of the famous orations and lectures, and of which he eventually became President. In 1873 he was honoured with the Presidency of the B.M.A. Queen Victoria made him a baronet, and he became her Sergeant-Surgeon, a post he held till his death in 1877 in his sixty-ninth year.

We are not told what Fergusson discovered on his visit. It was given out that the Emperor was suffering from rheumatism, and that diet and rest had been prescribed. We do know the Emperor was subject to attacks of what were called rheumatism, and which were held to be a legacy of the damp quarters he occupied during his long imprisonment. I would suggest however that there was another cause, well known to us nowadays, though it might not have been known then, and one arising from his amorous adventures. It is unlikely, to say the least, that a surgeon would have been summoned from London to treat rheumatism. Furthermore, Sir William, although a versatile general surgeon, was noted also for his uro-genital work, and with the benefit of hindsight I think we may safely deduce that the Emperor was suffering from some urinary disorder due to or associated with urethral stricture, and that Fergusson's visit provided a portent of things to come.

Since little more is known about Louis Napoleon's health for the next five or six years, may I, before leaving this first decade, mention some achievements for which he deserves credit.

In 1854, he took France into the Crimea in alliance with Britain, Turkey, and Sardinia to check the southward ambitions of Russia. The war ended in victory for the allies after appalling sufferings by the troops of both sides in the terrible Russian winter. A cynic has written that the aims and objects of the Crimean war were British, and the sacrifices and successes French. This is of course an exaggeration, even though the French did have four times as many troops engaged as we had, but it does seem true that only France came out of the war with military prestige intact. As for us, the lustre of British arms which forty years earlier had shone so brightly, was sadly dimmed, and it is doubtful if, on emergence from this war, our military prestige had ever been lower, despite the fame of two great cavalry charges.

The Crimean war was a triumph for France, and it enhanced the prestige of Louis Napoleon. In 1859, the Emperor joined forces with King Victor Emanuel of Sardinia in war against Austria, and crossed the Alps. Thanks largely to the genius of the French marshals Niel and McMahon, the Austrians were defeated in a number of battles, notably Solferino and Magenta, and the way was paved for that unification of Italy and its liberation from Papal as well as Austrian domination which were soon to come about. This was yet another triumph for France and the Emperor. An interesting side-light on the battle of Solferino is the inspiration it afforded a Swiss named Henri Dunant who was present on the field. Horrified by the inadequate care of the wounded, he wrote a book entitled " A souvenir of Solferino " which, widely publicised, aroused the world's conscience and which, together with his subsequent crusading, led to the Geneva Convention of 1864 and the rules which have for the most part been observed ever since by civilised nations. Dunant was one of the earliest recipients of the Nobel peace prize. Credit however is also due to Louis Napoleon for the support and encouragement he gave to Dunant.

Lastly, may I refer to a considerable achievement of a different kind. Towards the end of this decade, a remarkable transformation was effected in the face of Paris by a great French administrator, Georges Eugene Haussman, later Baron Haussman. Much of the centre of the city was cleared and replaced by magnificent boulevards, avenues, and squares. One writer has described the work as " The most successful and considerable piece of town planning ever to be carried out on the grand scale in an ancient city, and one which changed Paris from a mediæval city into a city of light and air ". Although it was the conception of Haussman to whom it is rightly a monument, it is also a monument to the Second Empire and to Louis Napoleon who had the vision to commission Haussman.

Thus far there has surely been little evidence of weakness or irresolution or lack of capacity. When the second half of his reign is considered however, there is a different story to tell, and an increasingly sombre one. Whether for the first time or not we cannot say, but we do know that the Emperor was catheterised for urethral stricture in 1861 by Felix Guyon, Professor of genito-urinary surgery at Paris. It would seem that thereafter this manipulation was carried out at intervals by the Emperor's personal physician, Dr. Conneau, whose devotion, it has been unkindly said, exceeded his skill with the catheter. There can be little doubt that in the course of time false passages were made and the bladder often infected.

Throughout the 1860's, Louis Napoleon's health steadily deteriorated, and as it did so his authority weakened. Criticism became more daring, and pressure on him to institute representative government and to restore lost freedoms ever stronger. In foreign affairs French influence was waning and France was meeting increasingly with rebuffs. In 1866 France yielded to pressure from the United States, gave up her dream of a French sphere of influence in America, and withdrew from Mexico. She left to his fate the Emperor Maximillian whom she had persuaded to ascend the Mexican throne three years earlier. A gentle and peaceable man, Maximillian thought he enjoyed the good will of the Mexican people, but he had really been maintained hitherto by French bayonets. With the French withdrawal revolutionaries speedily gained control. They captured Maximillian and shot him, while his wife, on a mission to Europe to seek help, went out of her mind and ended her days in an institution for the insane. The tragedy was only one of many which dogged the long life of the Emperor Francis Joseph of Austria-Hungary whose younger brother Maximillian was, and who died at the height of the Great War after a reign of sixty-eight years, one of the longest in world history. The affair was a sad and sorry one, and a blow to the prestige of Louis Napoleon. It was one of many during this decade, but the worst and culminating blow was yet to come.

In the closing years of the Empire, Louis Napoleon had become an ailing and weary man, while the French people now realised that all was not well with him. They had been getting suspicous because at times he had been compelled to absent himself from important functions, whilst at others which he did attend he had been observed to be in great distress.

We now know that famous specialists from the great medical centres of Europe had been secretly visiting the Emperor for some time past, and that whenever his indisposition could not be hidden he was always declared to be suffering from rheumatism. By 1870 it had become an open secret that he was suffering from a serious bladder complaint, and a well-known journalist could write in exasperation that he had never heard of the treatment of rheumatism by a catheter. Even at his best the Emperor was in considerable pain and found riding, or merely driving in a carriage, distressing. His urine was purulent and blood-stained, and he was subject to bouts of fever. One of his marshals, Clanrobert, has described how on one occasion he was by mistake admitted to the Emperor's presence and found him writhing in agony and consuming doses of laudanum. Something had to be done, and his personal medical attendants headed by the faithful Conneau called in consultation three famous French specialists. They were Nelaton, See, and Ricord.

Nelaton, perhaps France's leading surgeon, and the inventor of the flexible rubber catheter, had recently operated for stone on two famous people, and both had died as the Emperor well knew. And as Nelaton stood by the bedside and contemplated the possibilities, he felt far from comfortable. It is not surprising that the Emperor was hardly enthusiastic either.

Germain See was a brilliant young physician who had recently at the early age of thirty-two been made professor of medicine at the University of Paris.

Ricord was a noted venereologist and, where sexual morals were concerned, a most pronounced sceptic. Indeed a cynical contemporary has said that Ricord would have had no hesitation in prescribing mercury for the vestal virgins.

Unfortunately these eminent doctors differed, only Germain See insisting on the diagnosis of stone in the bladder and on the need for an exploration which he considered long overdue. This lack of unanimity was all that Louis Napoleon needed as an excuse to avoid an investigation which he dreaded. In the event, a period of rest in bed, a strict regime, and such remedial measures as were available effected sufficient improvement to enable him, unfortunately for France, to play his part in the tragedy which was about to involve her.

Briefly, the vacant Spanish throne had been offered to a Hohenzollern prince. Fearing Prussian influence on two main frontiers, France in uncompromising terms requested the Prussian king to withdraw the candidate. King William, somewhat surprisingly perhaps, courteously acceded to the request. Now comes a blunder which is almost incredible. Against responsible opinion and advice, the Emperor instructed his foreign minister, Gramont, to demand guarantees of such a nature that no self-respecting monarch could give them; and the king of course declined to do so. Instead of listening to the many voices of reason, the Emperor called and presided over a council of war, and after all too short a conference this sick man, mind clouded and judgment impaired by pain and toxæmia, decided on war and gave the order to mobilise. Nobody was better pleased than Bismarck whose dream of a united Germany was nearing fulfilment, and France was to find herself fighting not only a powerful Prussia flushed with recent successes, but a Prussia reinforced by the rest of the German kingdoms and principalities.

Louis Napoleon was a brave man, and he rode to war. From time to time riding in a carriage or even being jolted over the rough countryside in army wagons, but for the most part on horseback, sleepless and racked with pain, and often seen to be holding on to a tree or wagon wheel or being supported by aides whilst he faced the agony of trying to pass urine, he accompanied the army of his most famous marshal, McMahon. Years after his death his English physician, Sir William Gull, was to ask how any man in such a condition could have sat on a horse for so many hours.

Although her troops fought bravely and inflicted heavy losses on the Germans, France staggered from one disaster to another. McMahon, against his better judgment and over-ruled from Paris, was compelled to march to the relief of Metz, but he was caught at Sedan, virtually encircled, and forced to surrender with the Emperor and one hundred thousand men. When tendering his sword, Louis Napoleon is said to have expressed regret that he had not died on the battlefield, and this may well be so. What would have been regarded by Frenchmen as a hero's death might well have saved the dynasty, and the young Price Imperial, who was to be ambushed and killed by Zulus at Ulundi nine years later whilst serving as a British officer in a small colonial war, would have become the Emperor Napoleon the Fourth. But it was not to be. Three days after Sedan, a provisional republic was proclaimed in Paris, and the Emperor, now a prisoner in German hands, was deposed, whilst the Empress Eugenie fled to London.

The war continued for several months, and the end of hostilities did not come until after the fall of Paris following the very gallant defence of that city. France was humiliated, for in addition to paying a large indemnity and losing the provinces of Alsace and Lorraine she had had to suffer the indignity of seeing the Prussian king crowned first German Emperor in, of all places, the Hall of Mirrors at Versailles. Thus was sown one very important seed of the Great War. There followed the bloody episode of the Commune in which fifty thousand more Frenchmen lost their lives, and with its suppression the Third Republic came into being. Louis Napoleon was now freed by the Germans, and in January 1871 he rejoined his Empress in the village of Chislehurst in Kent. Here he lived out the short last phase of his life.

Thanks to a quiet and well ordered existence he was for the next eighteen months or so free from acute and urgent episodes, although never entirely free from pain and discomfort. It was too good to last however, and in October 1872 the Emperor was in serious trouble once again. He could scarcely walk and his constitutional and urinary signs and symptoms were back in full force. On the advice of Queen Victoria who befriended the exiled couple, Sir William Gull was asked to see the patient. It did not take Gull long to sum up the situation. He called in consultation Sir Henry Thompson, the well known urologist, and doubtless because their recommendations proved unpalatable to the Emperor, another celebrated surgeon, Sir James Paget, was called in. Before proceeding further with the story of the Emperor, may I say a few words about the careers of these three great doctors (Plates XXII, XXIII, XXIV).

William Gull was born in 1816, and he started out in life as an assistant school teacher, a precarious and poorly paid vocation in those days. Securing a minor appointment at Guy's Hospital, he so successfully combined his duties with those of a medical student that he qualified with the London M.B. at the age of twenty-five. His advance was rapid. Having in the meantime proceeded to the M.D., he was elected F.R.C.P. only seven years after qualifying, and three years later he was on the senior staff of Guy's. A brilliant lecturer and impressive public speaker, his remarkable powers of observation made him also a great clinician. I will refer to only two of his many original contributions to medical knowledge.

Gull had collaborated with Sutton of the London Hospital in a post-mortem study of the small arteries, at first in those dying from chronic Bright's disease, and later in those dying from other causes. They had described a condition which they had termed and which was long known as arterio-capillary fibrosis. Gull read a paper on their work to the London Clinical Society in 1872, and in it he made the striking statement that in many cases the arterial changes were primary and the renal secondary. He was on the track of what more than a generation later Clifford Allbutt was to call hyperpiesia, and the arterio-capillary fibrosis of Gull and Sutton was none other than the diffuse hyperplastic sclerosis which is part of the morbid anatomy of what we now call essential hypertension. The work was a notable pioneer effort in the story of arterial disease. A year later, again to the London Clinical Society, Gull read a paper to which he gave the very perceptive title of "A cretinoid condition in adults". It was the first clinical description of myxœdema.

Many distinctions came his way. He received honorary degrees from Oxford, Cambridge, and Edinburgh, and many fellowships of learned societies including the F.R.S. He was for many years Crown representative on the General Medical Council, and was the first London graduate to be a member of the Senate of London University. Although he was very prominent in the activities of the Royal College of Physicians, its highest office eluded him. Gull was a very forthright and independent man, and he was apt to be short and sharp with those from whom he differed, and they saw to it that he never became President. An example of his independent outlook was the contempt he had for the great variety of drugs by which most of his contemporaries set great store. Gull used only a few well tried ones, and the pharmaceutical blunderbuss, so beloved in his day, was not for him.

Following his successful treatment of the Prince of Wales for a severe attack of typhoid fever, Queen Victoria made him a baronet, and he eventually became her physician-in-ordinary. Highly successful in practice, he left over £300,000 when he died in 1890 at the age of seventy-four. William Gull was a product of a wonderful Guy's era which produced many great doctors, among them Richard Bright and Thomas Addison, and he suffers little if at all by comparison with them.

Henry Thompson was born in 1820, and he left the world of business at the age of twenty-four to enrol as a medical student at University College, of which his maternal grandfather, Samuel Smedley the artist, had been one of the founders. He qualified in 1850, gained the gold medal in surgery at the London M.B. in 1851, and the F.R.C.S. of England in 1853, the year in which he was elected assistant surgeon to University College Hospital only three years after qualifying. A year before he became a Fellow, he had been awarded the Jacksonian prize for his essay on " The pathology and treatment of urethral stricture ", thus early showing what was to be a life-long interest in genito-urinary surgery. And I may mention here that, eight years later, he achieved the unusual distinction of again being Jacksonian prize winner for a paper on " The healthy and morbid conditions of the prostate gland ". In due course, Thompson became professor of clinical surgery.

Whilst an assistant surgeon, he spent some time in Paris, studying the methods of Civiale who was the first to treat vesical calculus by crushing with the lithotrite, and whose custom it was to crush at repeated sittings and allow the patient to void the fragments in the intervals. On his return, Thompson, although he was not always successful as we shall see, improved on Civiale's technique by aiming at crushing the stone or stones and removing the fragments at one sitting. He did so by the use of the interesting armoury depicted in Plate XXV—a powerful lithotrite, a large evacuating catheter, and a special suction apparatus. And it does really bring a sense of relief to be assured that all this was done with the patient still under the anæsthetic. So great became Thompson's reputation that he was asked to operate for stone on King Leopold of the Belgians. He did so with success and was knighted by Queen Victoria.

International though his reputation was in his chosen calling, his fame rests almost as much on his other interests and accomplishments which were remarkably diverse. Thus he was an artist of merit who exhibited at both the Royal Academy and the Paris Salon, the author of two novels and numerous magazine articles all of which he himself illustrated, a competent astronomer with his own observatory, and a recognised authority on and noted collector of Nankin china. He was also for many years a champion of the cause of cremation and played a big part in bringing about its legalisation. Finally, no story of Henry Thompson is complete without reference to his famous " octaves " which were dinners of eight courses for eight people and held at eight o'clock. In later years he occasionally had one or two extra guests, but the dinners continued to be known as octaves, as the term had become so firmly established; and the accompanying illustration is of one of these exceptions (Plate XXVI). Ten diners are seated at a round table as usual, " to encourage the feast of reason and flow of soul ", and the dinner is in honour of the then Editor of the Lancet, Mr. Ernest Hart, who had just returned from a successful lecture tour of the United States. The other nine are all doctors and their names arouse nostalgic memories in those of us who are old enough to have lived in or near their times. Reading from left to right, they are Sir Richard Quain the celebrated anatomist, on whose works some of us were brought up. Next comes Sir James Paget, of whom more anon, and then Mr., later Sir Victor Horsley, the great University College Hospital pioneer in neuro-surgery who was to die tragically of heat stroke in Mesopotamia in 1916 whilst serving as consulting surgeon to the British forces. Facing us are Sir James Thompson and his guest of honour, Mr. Hart. Then come (with his back to us and in conversation with Horsley) Dr., later Sir George

Anderson Critchett, a noted ophthalmologist, and the eminent surgeon, Sir Thomas Spencer Wells of whose name successive generations of doctors need no reminding. Next, and half turned to us, is Sir William Henry Broadbent who gave his name to a well known clinical sign in adhesive pericarditis. Last but one, and just visible to us, is Mr., later Sir Joseph Fayrer, a surgeon who had had a distinguished career in India, while on the extreme right is Dr., later Sir Lauder Brunton, an Edinburgh graduate who came south to become a noted Bart's physician and a famous clinician and pharmacologist. Thompson gave more than three hundred of these octaves at roughly monthly intervals, and people famous not only in medicine but in many other walks of life attended them. When Prince of Wales, both King Edward the seventh and King George the fifth were guests on (I believe) more than one occasion. Sir Henry died in 1904 after an extraordinarily full life, and he was of course cremated. He was nearly eighty-four.

James Paget was born at Great Yarmouth in 1814 the son of a shipowner and one of seventeen children. Educated privately, he was apprenticed at the age of sixteen to a well known local surgeon, one Charles Costerton who had been trained at St. Bartholomew's Hospital and was surgeon to the Yarmouth Hospital. He was a diligent apprentice, and during his four years with Costerton he collaborated with his brother Charles in writing a book on the flora and fauna of Great Yarmouth and the surrounding countryside, a book which sold for half-a-crown and brought in some much needed money, for his father had got into financial difficulties as a result of the slump in the shipping trade following the wars with Napoleon. At the age of twenty he was accepted as a medical student at St. Bartholomew's Hospital, and straightaway, as was usual at that time, began to dissect the human body. It was towards the end of his first term that he noticed in the voluntary muscles of the part he was dissecting, tiny white specks which, with the aid of a borrowed microscope he showed to be cysts containing worms. A famous anatomist, Sir Richard Owen, identified the worm as a nematode and named it; but the credit for the discovery of the *Trichinella spiralis* belongs to one who was at the time but a first term medical student. Paget read a paper on the subject to the Abernethian Society at Bart's on February 6th, 1835, and the minutes of that meeting make interesting reading and are a pointer to the great career he was to have. In 1836 Paget was admitted M.R.C.S., and if it now appears strange that he should have been able to qualify in only two years, it has to be remembered that his four years' apprenticeship to a capable doctor had given him a training and experience which were of particular value in an age when methods and requirements were vastly different from those of to-day.

It was in 1843 that the Royal College of Surgeons of England instituted its Fellowship. It celebrated the occasion by electing three hundred surgeons to the new status, and Paget was one of them. Three years later he was elected, in the face of tough opposition, to the senior surgical staff of St. Bartholomew's Hospital as assistant surgeon. A master of concise and lucid English, he was a superb lecturer and moreover an accomplished public speaker to whom no less a person than Mr. Gladstone paid tribute. Paget wrote widely and made many original contributions to knowledge. I have already referred to one; I shall mention just two more.

We are all familiar with that well known eczema-like state of the nipple and areola which is so often the precursor and indeed sometimes the accompaniment of duct carcinoma of the breast. This condition was first recognised and described by Paget, and it has been known ever since as Paget's disease of the nipple. At least equally familiar to us is that disease of the bones in which, on account of increasing size of the calvarium, the sufferer needs to take a progressively larger size in hats, and as a result of kyphosis and bowing of the thigh and leg bones, loses height. This condition was first described by Paget, is of course osteitis deformans, and in my day was, as often as not, referred to as Paget's disease of the bones. Paget will surely be remembered for many reasons, but if all else about him is forgotten the two conditions I have just referred to will perpetuate his name.

Honorary degrees and fellowships were showered upon him, and he held many high offices. Thus, he became President of the Royal College of Surgeons, was President of the great International Congress of Medicine held in London in 1881, and for twelve years was Vice-Chancellor of London University. On the death of Sir William Fergusson he was appointed Sergeant-Surgeon to Queen Victoria who had earlier made him a baronet, and with all this and much more he found time to conduct perhaps the biggest private surgical practice in London. Paget was a gentle, modest, courteous man and essentially of a retiring disposition, and he became a prominent figure really in spite of himself. He died in 1899 when just short of his eighty-sixth birthday, and a memorial service was held in Westminster Abbey.

And now back to the Emperor! Gull, Thompson, and Paget agreed on the diagnosis of stone in the bladder and its associations, but whereas Gull and Thompson were for immediate operation Paget took a conservative line and counselled deferment in the hope of some improvement in the patient's general condition. So two months went by before Louis Napoleon was brought to the operating table. On Jan. 2nd, 1873, he was anæsthetised by a Mr. Clover, described as the leading chloroformist of the day. Thompson passed the lithotrite, seized and crushed a phosphatic stone the size of a walnut, and removed as many fragments as he could.

The Emperor passed the next twenty-four hours tortured by the passage of calculous fragments, and then, owing to complete obstruction, he was again chloroformed. A large fragment was found to be blocking the membranous urethra and after manipulation with a probe was moved sufficiently to permit the introduction once again of the lithotrite to remove this and other fragments. There followed four more days of torment, and then the prostatic urethra became obstructed. Yet a third time was the Emperor chloroformed and the fragment probed back in to the bladder to permit the flow of what might be called urine, but was in truth little more than blood and pus. To quote the subsequent report, " No further steps were taken owing to the extreme irritability of the parts ", and I think this laconic observation provides a sufficient indication of the sufferings of the wretched man.

On the morning of January 9th, exactly one week after his first operation, Louis Napoleon seemed easier and brighter, so much so indeed that a conference was held and the decision taken to do a fourth exploration at noon. So optimistic did everybody become that one of the Emperor's aides named Rohet remarked " The Emperor is much better and will soon be passing water as well as I can ", an observation which was scarcely enlighten-

Plate XXII—Sir William Withey Gull (1816-1890). Portrait from a photograph lent by Guy's Hospital.

Copyright : Guy's Hospital

Plate XXI—Sir William Fergusson (1880-1877). From a photogravure by the Swan Electric Engraving Company of the picture by Rudolf Lehmann. Impression in the Wellcome Historical Medical Museum and Library. By courtesy of the Wellcome Trustees.

Plate XXIV—Sir James Paget (1814-1899). From an original photograph by Barraud in the Wellcome Historical Medical Museum and Library. By courtesy of the Wellcome Trustees.

Plate XXIII—Sir Henry Thompson (1820-1904). From an original photograph by Lock and Whitfield. In the Wellcome Historical Medical Museum and Library. By courtesy of the Wellcome Trustees.

Plate XXV—Lithotrite, suction apparatus and evacuating catheter. Specimens in the Wellcome Historical Medical Museum and Library.

Copyright : Wellcome Historical Medical Museum and Library. By courtesy of the Wellcome Trustees.

Plate XXVI—A special 'Thompson's Octave' for Mr. Ernest Hart. From a picture of 1894 by Solomon J. Solomon. In the Wellcome Historical Medical Museum and Library. Copyright : Wellcome Historical Medical Museum and Library. By courtesy of the Wellcome Trustees.

ing, since no one knew what Rohet's *own* abilities were in this respect. At 10.30 however, Louis Napoleon's condition suddenly and dramatically worsened, and in a few minutes he was dead.

A post-mortem was performed by the celebrated morbid anatomist, Burdon Sanderson, and attended by all the doctors concerned. As we should expect, the urinary bladder was found to be the seat of long-standing cystitis and to contain calculous fragments. The ureters were greatly hypertrophied, the left being nearly as thick as the aorta, and both renal pelves were very dilated. The left kidney was completely disorganised and functionless, while the right kidney was badly damaged and what functioning tissue remained was the seat of acute inflammation. It is surprising that no other abnormalities were found elsewhere in the body, and one may wonder what a modern pathologist would have had to report. However, the cause of death was given as uræmia, and there will be few who will quarrel with that verdict.

The report was published in the medical journals and in the Times, and it was signed by all except Gull who published his own report independently. Why he did so is not clear, for it differed but slightly from the official one. Such action was characteristic of Gull, and one can understand why he was unpopular with many of his colleagues.

With the publication of the reports, a storm of criticism broke out on both sides of the Channel, but it was particularly violent in the French press where it was directed at the Emperor's English doctors. If an anæsthetic were necessary the Emperor should have had ether, not chloroform, they said; and again, that his strength had been sapped by over-sedation with drugs. Even was it said that the Emperor was in no fit state to be operated on at all. Conveniently forgotten were the years when he could and should have had radical treatment. It is obvious that most of the criticisms were spiteful and ill-informed. May I quote what the Times had to say in a leader:—"It is melancholy to think that, notwithstanding all our discoveries and all our progress, several celebrated physicians and surgeons are still compelled to put their hands to a confession that the disease of the kidneys which must so soon have killed the Emperor, existed to a degree which was not suspected, and, if it had been suspected, could not have been ascertained. A man may still, it appears, die under the hands of the first doctors of the world, of a great organic disease without their knowledge, and the only reflection which is not painful in this agonising dissection of human infirmities is that he who endured them now endures them no longer".

It is true of course that Louis Napoleon could not have survived much longer in any case. It is also true that thirty years had passed since Richard Bright, a Bristolian, had described the renal conditions which bear his name, and that many causes and consequences of renal disease were already well-known. It is hard to believe that men of the calibre of Gull, Thompson, and Paget were not aware of the danger of renal failure, and therefore it does seem odd that none of them appears at any time to have warned of the likelihood of such an event. Nevertheless, even with this reservation, the Times's verdict is surely harsh and unjustified. These eminent doctors acted in accordance with the best practice of the day, crude though it may appear to us now, and if there had to be criticism surely it should have been reserved for those who, many years before, failed to realise or to convince the Emperor of the need of radical treatment.

There have been doctors who have not hesitated to speak bluntly, even to kings. Less than thirty year later, a great London Hospital surgeon, Frederick Treves, was to have the unenviable task of informing King Edward the Seventh, only two days before the King's coronation, that an immediate operation was imperative for what was described in the language of the day as inflammation of the vermiform appendix. When the King protested that all arrangements had been made and he must go to his coronation, Treves replied, "Then Sir, you will go in your coffin". Whether or not Treves believed literally what he said I do not know, but it certainly had the desired effect. As we all know, the King's coronation was postponed to the great inconvenience of all sorts of people and interests, the King had a successful operation, and he lived for a further nine years.

It is tempting to wonder what the course of history might have been if Louis Napoleon, before he had suffered irreversible and irreparable damage, had had the benefit and acted on the advice of a Treves, and if a vigorous and mentally alert man had been at the helm of France in 1870. In such an event, might we still have been living in a world which, for all its many hazards was a safer one, and with all its many defects surely a more gracious one? Further, might posterity's assessment of Louis Napoleon have been kinder? Speculating on what might have been, though fascinating, is not very profitable, and there is of course no answer.

## Figures and Tables

**Plate IXX f1:**
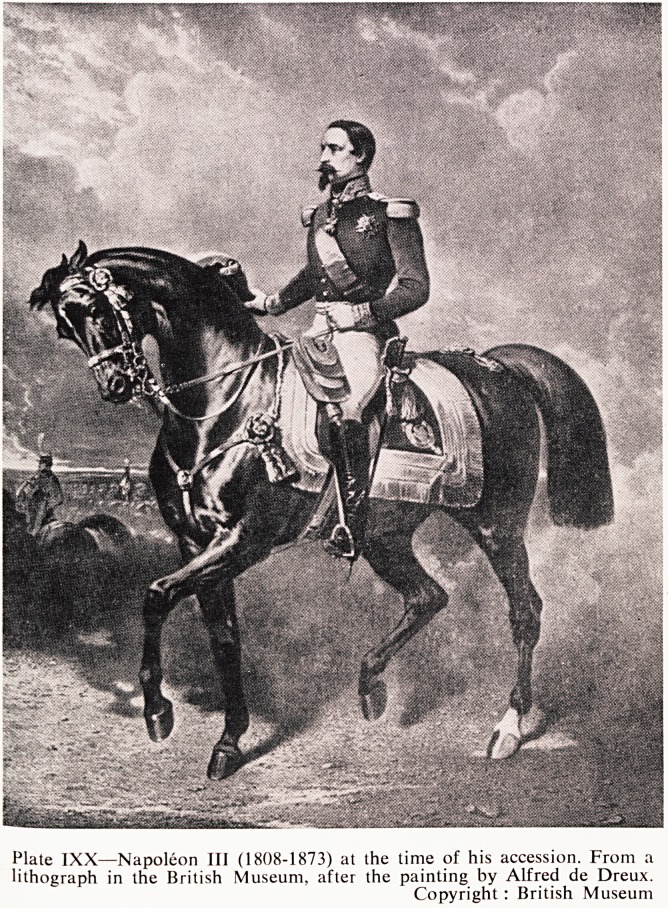


**Plate XX f2:**
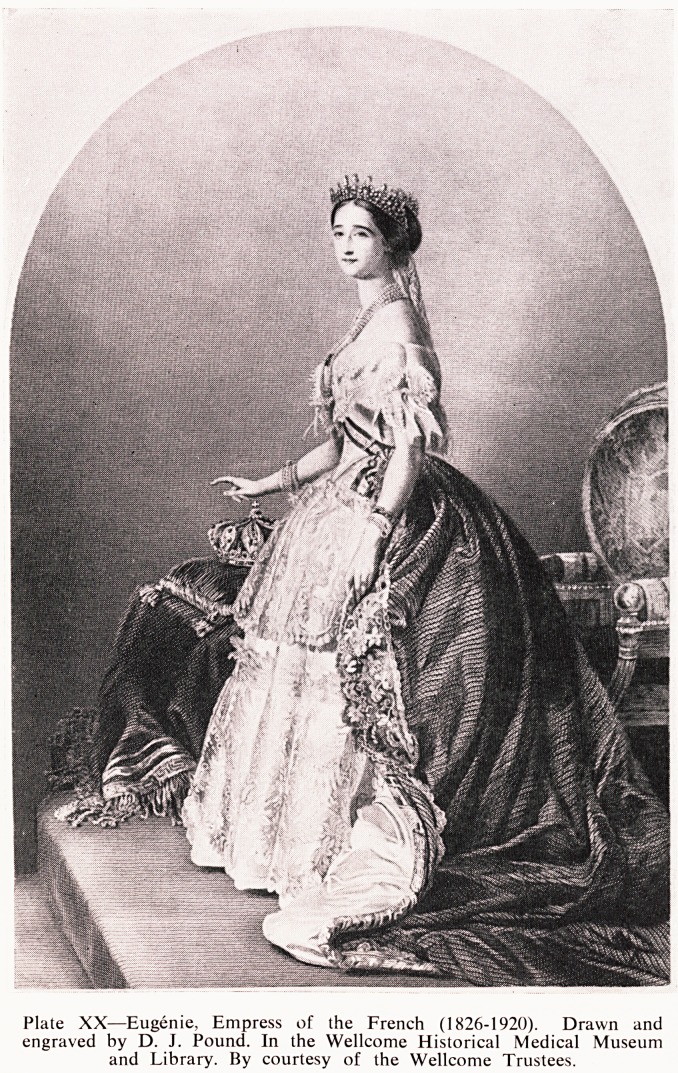


**Plate XXI f3:**
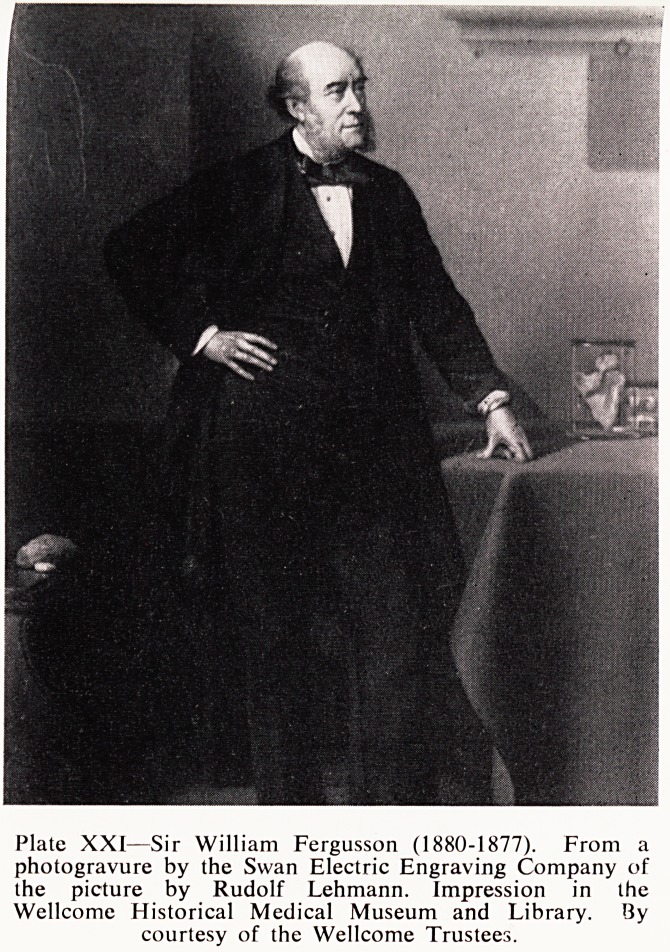


**Plate XXII f4:**
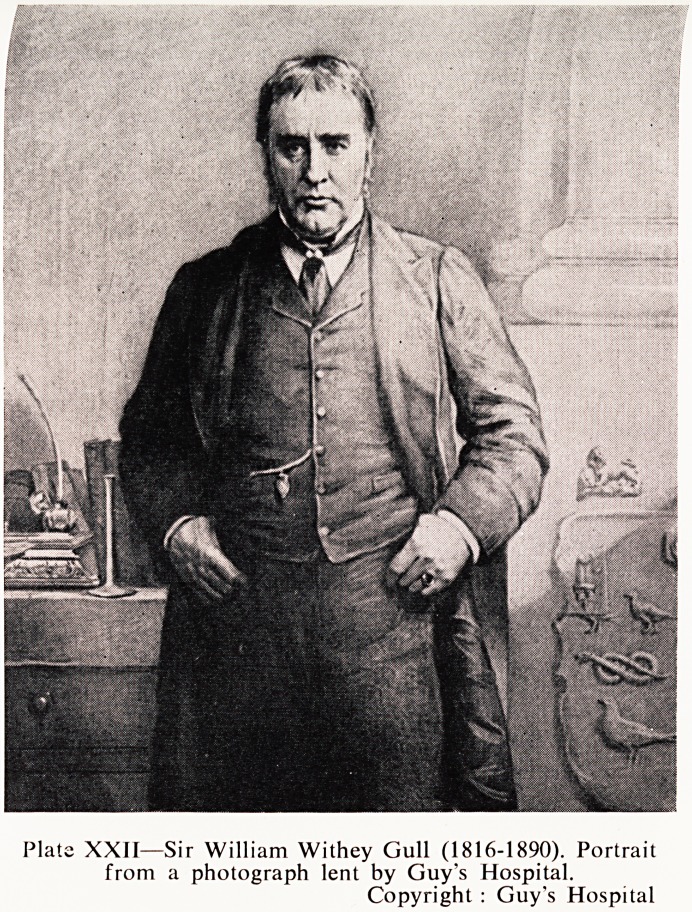


**Plate XXIII f5:**
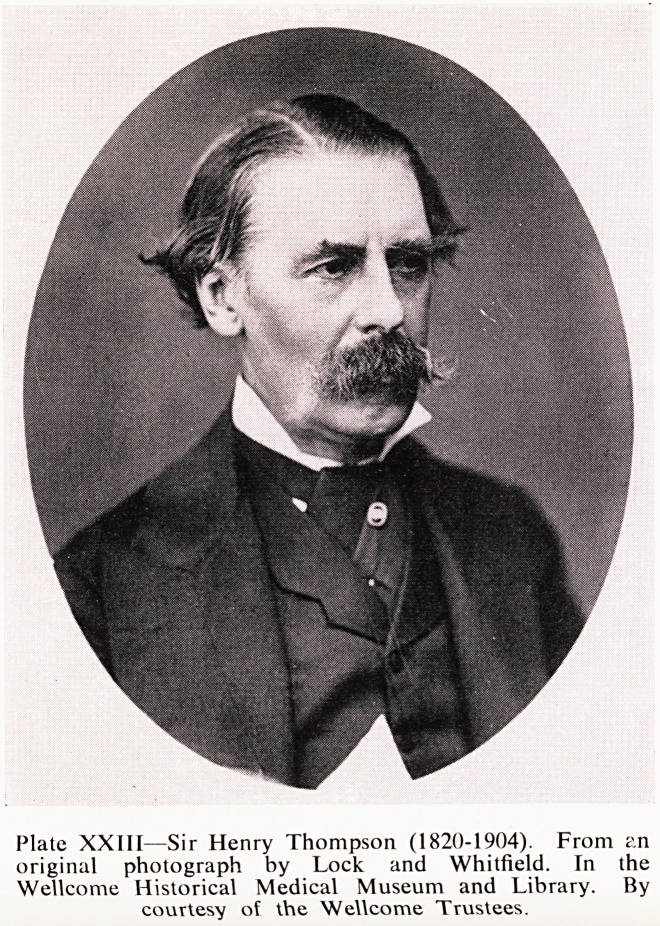


**Plate XXIV f6:**
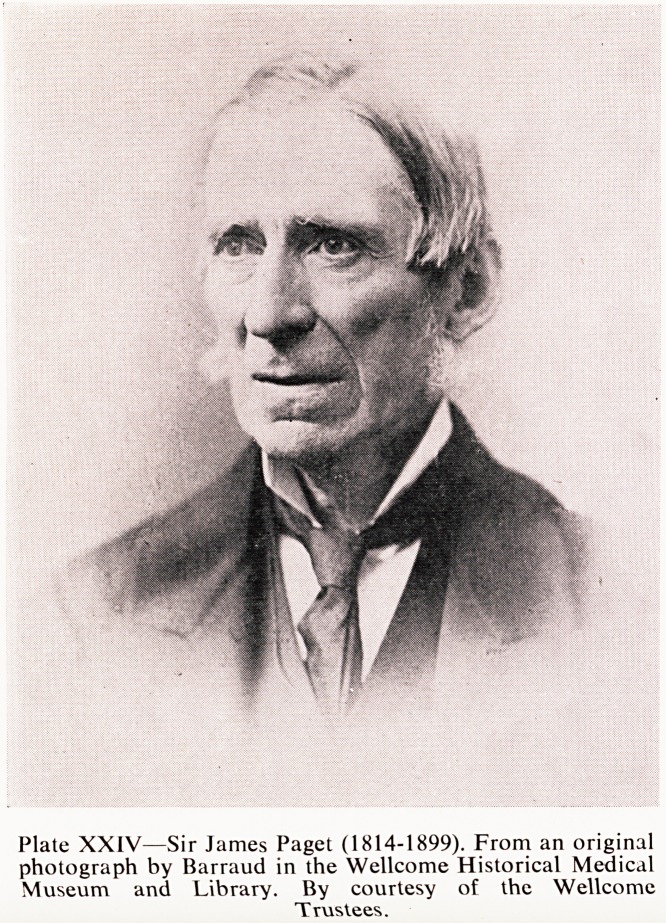


**Plate XXV f7:**
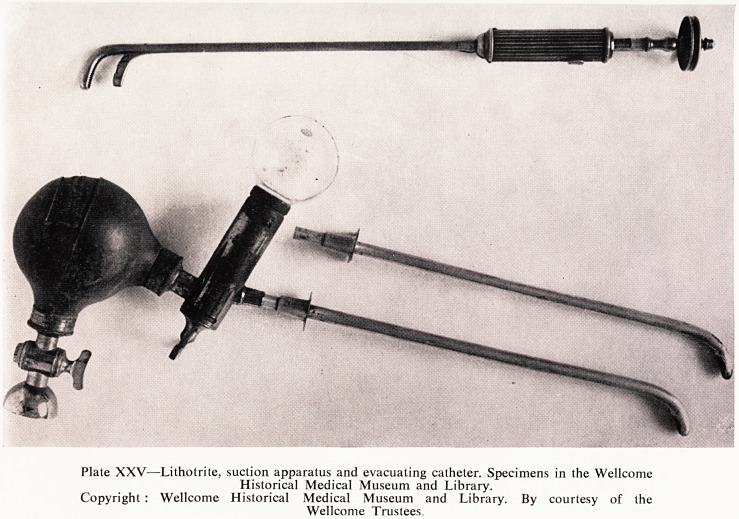


**Plate XXVI f8:**